# Inhibition of Pancreatic Lipase and Triacylglycerol Intestinal Absorption by a *Pinhão* Coat (*Araucaria angustifolia*) Extract Rich in Condensed Tannin

**DOI:** 10.3390/nu7075242

**Published:** 2015-07-09

**Authors:** Roselene Ferreira Oliveira, Geferson Almeida Gonçalves, Fabíola Dorneles Inácio, Eloá Angélica Koehnlein, Cristina Giatti Marques de Souza, Adelar Bracht, Rosane Marina Peralta

**Affiliations:** 1 Department of Biochemistry, State University of Maringá, Maringá 87020900, Brazil; E-Mails: oliveiraroselene@hotmail.com (R.F.O.); gfersonag@ig.com.br (G.A.G.); cgmsouza@uem.br (C.G.M.S.); adebracht@uol.com.br (A.B.); 2 Instituto Federal do Paraná, Jacarezinho 86400000, Brazil; E-Mail: fabiola.inacio@ifpr.edu.br; 3 Department of Nutrition, Federal University of the South Border, Realeza 85770000, Brazil; E-Mail: eloa-angelica@hotmail.com

**Keywords:** tannins, lipase, enzyme, obesity

## Abstract

The purpose of the present work was to characterize the possible inhibition of pancreatic lipase by a tannin-rich extract obtained from the *pinhão* (*Araucaria angustifolia* seed) coat, based on the previous observation that this preparation inhibits α-amylases. Kinetic measurements of pancreatic lipase revealed that the *pinhão* coat tannin is an effective inhibitor. Inhibition was of the parabolic non-competitive type. The inhibition constants, ${\bar{K}}_{i1}$ and ${\bar{K}}_{i2}$, were equal to 332.7 ± 146.1 μg/mL and 321.2 ± 93.0 μg/mL, respectively, corresponding roughly to the inhibitor concentration producing 50% inhibition ([I]_50_). Consistently, the *pinhão* coat extract was also effective at diminishing the plasma triglyceride levels in mice after an olive oil load; 50% diminution of the area under the plasma concentration *versus* the time curve occurred at a dose of 250 mg/kg. This observation is most probably the consequence of an indirect inhibition of triglyceride absorption via inhibition of pancreatic lipase. For the *pinhão* coat tannin, this is the second report of a biological activity, the first one being a similar inhibition of the absorption of glucose derived from starch as a consequence of an inhibitory action on α-amylases. Taken together, these effects represent a potential anti-obesity action, as suggested for other polyphenol or tannin-rich preparations.

## 1. Introduction

Proanthocyanidins are the most common group of flavonoids in the Western diet. They are also known as condensed tannins, because they comprise a group of polyhydroxyflavan-3-ol oligomers and polymers of flavonol subunits linked by carbon to carbon bonds [1]. They have attracted great attention due to rapid growing evidence associating these compounds with a wide range of potential health benefits [2,3]. The opinion has been frequently expressed that the discovery of new materials rich in tannins with enzyme inhibitory properties could contribute to the development of new drugs useful in the control and treatment of diabetes, obesity and other physiological disorders [2,3]. *Pinhão* is the name generally given to the seed of the coniferous tree *Araucaria angustifolia* [4], which was once quite abundant in southern Brazil and which is still an important component of its flora. The *pinhão* seed is largely consumed as a food, but the coat is generally discarded. The latter, however, is rich in condensed tannins, belonging predominantly to the procyanidin class, which are chains of catechin, epicatechin and their gallic acid esters [5]. With respect to the *pinhão* coat tannins, it has been shown that an extract rich in these compounds (90% of its weight) is able to inhibit both salivary and pancreatic α-amylases [5]. As a consequence of these actions, the *pinhão* coat extract is also effective at diminishing the post-prandial glycemic levels in rats after starch administration. For these reasons, it was suggested that the *pinhão* coat extract could be used as an adjuvant in the suppression of postprandial hyperglycemia in diabetic patients [5].

The action of tannins or polyphenols in general is not restricted to the α-amylases or other types of glucosidases. Inhibition of these classes of enzymes is generally also associated with the inhibition of lipases, especially pancreatic lipase [6,7].It has been shown, for example, that a polyphenol preparation from the *Acacia mearnsii* bark is able to inhibit pancreatic lipase in addition tothe glucosidases maltase and sucrase [7]. By virtue of this observation and considering our previous finding of an inhibition of α-amylases by the *pinhão* coat extract rich in tannins [5], the logical hypothesis that can be formulated is that the tannins of the *pinhão* coat are equally able to inhibit pancreatic lipase or even other lipases. If this hypothesis is correct, the *pinhão* coat tannins should be equally able to affect lipid absorption *in vivo* [8]. Testing of these two hypotheses was precisely the purpose of the present work, which consisted of both *in vitro* experiments with the porcine pancreatic lipase and *in vivo* experiments with mice loaded intragastrically with olive oil. The results should contribute further to clarifying the potential usefulness of the *pinhão* coat, which is otherwise merely discarded as a waste product.

## 2. Experimental Section

### 2.1. Materials

Porcine pancreatic lipase (Type II) and orlistat were purchased from Sigma-Aldrich Co. The *A. mearnsii* bark tannin was purchased from Labsynth, Brazil. All other reagents were of the highest available grade.

### 2.2. Animals

Male healthy Swiss mice (35 g to 40 g, on average 4 weeks old) were used in all experiments. Each mouse was kept at room temperature (22 ± 1 °C) and humidity with a 12-h light/dark cycle. The experiments were approved by the Ethics Committee of Animal Experimentation of the University of Maringá.

### 2.3. Preparation of the Pinhão (A. angustifolia) Coat Extract

*Pinhão* seeds used in this study were purchased at a local market (Maringá, PR, Brazil) and prepared according to methods described elsewhere [5]. Briefly, the seeds used in this work were washed with tap water and dried at room temperature for 24 h. The coats of the seeds were removed and dried at 40 °C until constant weight. The seed coats corresponded to approximately 30% of the total weight. After drying, the seed coats were milled into a fine powder. The powder (100 g) was mixed with 300 mL of 70% ethanol (in water) at room temperature and maintained under agitation at 140 rpm for 3 h. The extractions were repeated three times. No increases in yield were achieved by further extractions. The combined mixtures were filtered through Whatman filter paper Number 1 and concentrated with a rotary vacuum evaporator at 40 °C to eliminate ethanol and finally freeze dried.

### 2.4. Pancreatic Lipase Activity and Kinetics

The porcine pancreatic lipase was assayed using *p*-nitrophenyl-palmitate as the substrate and spectrophotometrically recorded at 410 nm [9]. The substrate solution was prepared by suspending 20 mg of *p*-nitrophenyl palmitate in 10 mL of isopropanol. The suspension was sonicated until complete dissolution of *p*-nitrophenyl-palmitate. At the time of use, this stock solution was diluted with isopropanol to concentrations up to 0.5 mg/mL. The porcine pancreatic lipase was dissolved in Tris-HCL buffer (pH 8.0) at the concentration of 2 mg/mL. This suspension was centrifuged at 2000× *g* for five minutes and the supernatant used as the source of enzyme. The reaction mixture (2.4 mL) contained 100 mM Tris-HCl buffer (pH 8.2) and 530 μM substrate and was 25% isopropanol. After pre-warming the reaction mixture at 37 °C, the enzyme solution was added (0.1 mL), and the incubation was continued for 20 min at the same temperature. The reaction was stopped by transferring the reaction vessel to a bath of boiling water. After 10 min, the incubation was cooled to room temperature and centrifuged at 1500× *g* for 5 min. Absorbance of the supernatant at 410 nm was determined against a blank solution containing denatured enzyme. One enzyme unit was defined as 1 µmol of *p*-nitrophenol enzymatically released from the substrate per minute per mL. The kinetic measurements were done in the same way as described for the inhibition assays, except that the substrate concentration was varied by diluting the stock solution with isopropanol to the desired concentration, so that the isopropanol concentration was the same in each assay.

### 2.5. Oral Olive Oil Tolerance Tests in Mice

The intestinal absorption of triglycerides was tested by means of an oral olive oil tolerance test in mice [7]. The mice were deprived of food for 18 h before the experiment. *Pinhão* coat extract solutions were administered orally at doses of 100 mg, 250 mg and 500 mg per kg body weight. Olive oil was subsequently administered orally (5 mL per kg body weight). Before and at 1.5 h, 3.0 h, 4.5 h and 6.0 h after olive oil administration (or distilled water for the controls), blood samples from the tail vein were analyzed by means of an Accutrend Plus Roche triglycerides meter.

Seven groups of mice (*n* = 3 per group) were utilized: (1) the positive control, only intragastric olive oil (5 mL per kg) administration; (2) the negative control, only tap water administration; (3) intragastric administration of olive oil plus orlistat (50 mg/kg); (4) intragastric administration of olive oil plus *A. mearnsii* tannin (500 mg/kg); (5) intragastric administration of olive oil plus 100 mg/kg *Pinhão* extract; (6) intragastric administration of olive oil plus 250 mg/kg *Pinhão* coat extract; and (7) intragastric administration of olive oil plus 500 mg/kg *Pinhão* coat extract.

### 2.6. Calculations and Statistics

Statistical analysis of the data was done by means of the Statistica program (Statsoft, 1998, Tulsa, OK, USA). Fitting of the rate equations to the experimental initial rates was done by means of an iterative non-linear least-squares procedure using the Scientist software from MicroMath Scientific Software (Salt Lake City, UT, USA). The procedure requires the introduction of preliminary estimates of each parameter. These preliminary estimates are improved by each successive iteration in which the squared difference between the calculated and experimental data is progressively diminished until it converges towards a minimum [10,11].

The decision about the most adequate model (equation) was based on the model selection criterion (MSC) and on the standard deviations of the optimized parameters. The model selection criterion, which corresponds to the normalized Akaike information criterion [12], is defined as:

(1) $$\text{MSC}=\text{ln}\left[\frac{\sum_{i=1}^{n}w_{i}{\left(Y_{{\text{obs}}_{i}}-{\bar{Y}}_{\text{obs}}\right)}^{2}}{\sum_{i=1}^{n}w_{i}{\left(Y_{{\text{obs}}_{i}}-Y_{{\text{cal}}_{i}}\right)}^{2}}\right]-\frac{2p}{n}$$

Y_obs_ are the experimental reaction rates, ${\bar{Y}}_{\text{obs}}$ the mean experimental reaction rate, Y_cal_ the theoretically calculated reaction rate, w the weight of each experimental point, n the number of observations and p the number of parameters of the set of equations. In the present work, the model with the largest MSC value was considered the most appropriate, provided that the estimated parameters were positive.

## 3. Results and Discussion

### 3.1. Concentration Dependence of the Lipase Inhibition

**Figure 1:**
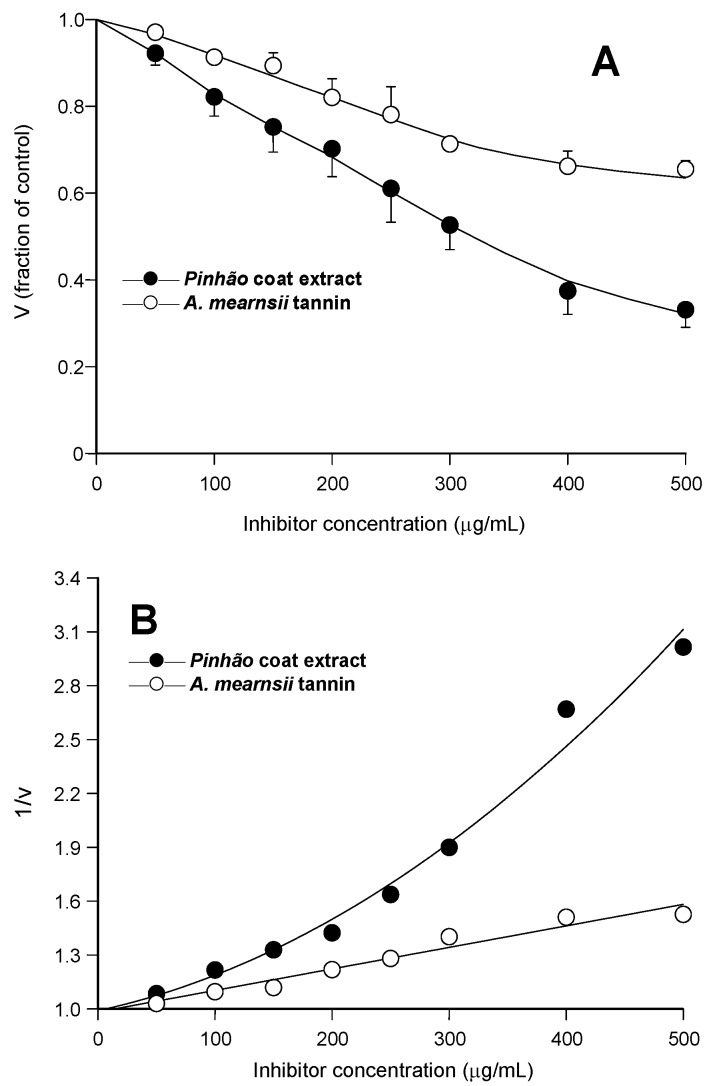
Inhibition of pancreatic lipase by *pinhão* coat extract and *A. mearnsii* tannin: concentration dependences. Initial reaction rates were measured as described in the Experimental Section. Each datum point represents the mean of three independent determinations: (**A**) relative reaction rates (v); (**B**) inverse reaction rates (1/v). The continuous lines in (B) represent the regression curves that were calculated after parabolic (A) and linear (B) regression analysis. The optimized equation for the *pinhão* coat extract was: y = 0.989 + 0.00141·x + 0.00000567·x^2^ (*r* = 0.992); for the *A. mearnsii* tannin: y = 0.982 + 0.00119·x (*r* = 0.981).

In the first experiments, the hypothesis of an inhibitory action of the *pinhão* coat extract on pancreatic lipase was investigated by measuring the enzyme activity at a fixed substrate concentration (530 μM *p*-nitrophenylpalmitate) and varying concentrations of the extract (up to 500 μg/mL). Parallel experiments were run with the *A. mearnsii* tannin (up to 500 μg/mL), as there is previous evidence that an *A. mearnsii* polyphenol preparation inhibits pancreatic lipase activity [7]. In Figure 1A, the relative rates were represented against the inhibitor concentration. It is apparent that the *pinhão* coat extract inhibited the enzyme with a clear concentration dependence, reaching 68% inhibition at the concentration of 500 μg/mL. The inhibition by the *A. mearnsii* tannin was reproduced in our experiments, with 35% inhibition at the concentration of 500 μg/mL. This is very close to the inhibition degree reported previously [7], but clearly less pronounced than that observed with the *pinhão* coat extract. Another difference between the actions of the *pinhão* coat extract and the *A. mearnsii* tannin is revealed by Figure 1B, in which the reciprocals of the reaction rates were represented against the respective concentrations. Whereas the inhibition caused by the *A. mearnsii* tannin was of the linear type, that caused by the *pinhão* coat extract was clearly parabolic. This has mechanistic implications and must be taken into account when further analyzing the inhibition caused by the *pinhão* coat extract in kinetic terms [13,14].

### 3.2. Kinetics of Pancreatic Lipase Inhibition

**Figure 2:**
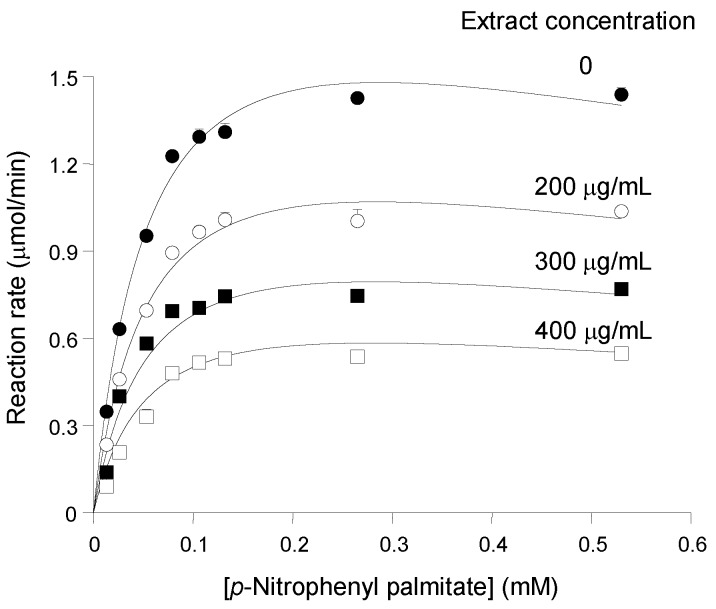
Substrate and inhibitor concentration dependences of the reaction rates of pancreatic lipase. Initial reaction rates, measured as described in the Experimental Section, were represented against the substrate concentration. The *pinhão* coat extract concentration in the assay medium is given on each curve. Each datum point represents the mean of at least three measurements. The error parameters are mean standard errors, which are not visible when smaller than the symbol size. The continuous lines represent the theoretical curves calculated according to Equation (3) with the optimized parameters listed in Table 1.

In order to characterize further the inhibition of pancreatic lipase by the *pinhão* coat extract, the activity of the enzyme was measured at different substrate and inhibitor concentrations. The results are summarized in Figure 2, which shows saturation curves that were progressively lowered as the *pinhão* coat extract was added at progressively increased concentrations. The saturation curves do not show any tendency of convergence at high substrate concentrations, which excludes the possibility of competitive inhibition. On the other hand, the saturation curve of pancreatic lipase in the absence of the inhibitor does not obey the simple Michaelis—Menten equation. This is revealed by the fact that the reaction rate ceased to increase when the substrate concentration was increased from 130 μM to 530 μM (Figure 1), revealing an apparent premature saturation. This particularity results in non-linear Lineweaver—Burk plots (not shown) with a concave up curve near the 1/v axis [13,14]. The latter phenomenon has been generally interpreted as substrate inhibition. Although it is not very pronounced in the concentration range investigated in the present work, it must be taken into account when analyzing the kinetics of the inhibition. Furthermore, consideration must also be given to the fact that the inhibition caused by the *pinhão* coat tannin was parabolic. This kind of relationship means that more than one inhibitor molecule binds to a single site in the enzyme, enhancing inhibition at higher concentrations to degrees that are above those expected when a single molecule binds to the site [13,14]. In principle, one may assume that the following complexes are formed in the presence of enzyme (E), substrate (S) and inhibitor (I): ES, ES_2_, EI, ESI, ES_2_I, EI_2_, ESI_2_ and ES_2_I_2_. If one assumes that (1) only the complex ES leads to product formation (*i.e.*, all other complexes are inhibitory), (2) binding of the second substrate molecule to the enzyme does not change its affinity to the inhibitor, (3) steady-state conditions hold for the substrate to enzyme interactions and that the (4) enzyme to inhibitor interactions are in equilibrium, the following equation can be derived for the reaction rate (v) dependence from both substrate ([S]) and inhibitor ([I]) concentrations [13,14]:

(2) $$v=\frac{V_{\max}[S]}{K_{M}\left(1+\frac{\left[I\right]}{K_{i1}}+\frac{{\left[I\right]}^{2}}{K_{i1}K_{i1}^{\prime }}\right)+[S]\left(1+\frac{\left[S\right]}{K_{iS}}\right)\left(1+\frac{\left[I\right]}{K_{i2}}+\frac{{\left[I\right]}^{2}}{K_{i2}K_{i2}^{\prime }}\right)}$$

V_max_ is the maximal reaction rate, K_M_ the Michaelis constant and K_iS_ the substrate inhibition constant; K_i1_ and K’_i1_ are the inhibition constants for the EI andEI_2_ complexes, respectively; K_i2_ the inhibition constants for the ESI and ES_2_I complexes; and K’_i2_ the inhibition constant for the ESI_2_ and ES_2_I_2_ complexes. These inhibition constants may be true dissociation constants under certain limiting conditions, but especially if more than one inhibitor is present in the *pinhão* coat extract, they are complex functions of several individual dissociation constants, but are still a measure of the potency of the inhibition [13,14]. It should be noted that the presence of more than one inhibitor does not invalidate Equation (2), provided that their concentrations are kept at constant ratios, as occurs when different amounts of the same extract are added [13,14]. Equation (2) was fitted to the whole experimental data set shown in Figure 2. Fitting was successful in that convergence between experimental and calculated data was reached quickly and positive values were obtained for all parameters. Table 1 lists the parameters obtained, which reveal consistent values for K_M_, V_max_ and K_iS_ with relatively low standard deviations. The optimized values of the inhibition constants, however, indicate that Equation (2) might not be a good description of the data. Firstly, the standard deviations of the four inhibition constants greatly exceed their optimized values, meaning that they cannot be determined with an acceptable precision. Moreover, the value of K_i1_ is exceptionally high, and the standard deviation exceeds it by orders of magnitude. Clearly, K_i1_ cannot be determined. This happens if the concentration of the complex EI is too low to be detected, which renders the [I]/K_i1_ term in Equation (2) non-significant. The same applies to the K_i2_ value, which greatly reduces the significance of the [I]/K_i1_ term in Equation (2) and also suggests that the concentrations of the ESI and ES_2_I complexes are low. An alternative to Equation (2) would be Equation (3) in which binding of at least two molecules of the inhibitor is considered to occur practically at a single step, so that only binary complexes of the inhibitor with the various enzyme forms are considered, namely EI_2_, ESI_2_ and ES_2_I_2_ [15]:

(3) $$v=\frac{V_{\max}[S]}{K_{M}\left(1+\frac{{\left[I\right]}^{2}}{{\left({\bar{K}}_{i1}\right)}^{2}}\right)+[S]\left(1+\frac{\left[S\right]}{K_{iS}}\right)\left(1+\frac{{\left[I\right]}^{2}}{{\left({\bar{K}}_{i2}\right)}^{2}}\right)}$$

In Equation (3), ${\bar{K}}_{i1}$ and ${\bar{K}}_{i2}$ are composite dissociation constants for the combination of at leasttwo inhibitor molecules with free enzyme or with substrate-complex enzyme, respectively. Fitting of Equation (3) produced more realistic parameters as revealed by the second column in Table 1. Figure 2, in turn, allows a graphical comparison of the experimental and calculated points, as the continuous lines were calculated according to Equation (3) using the optimized parameters. The K_M_, V_max_ and K_iS_ values were close to those found when fitting Equation (2), but with smaller standard deviations. Equation (3) is no doubt a better description of the experimental data, as revealed by the smaller sum of squared deviations and greater model selection criterion, which are also given in the last two lines of Table 1. The ${\bar{K}}_{i1}$ and ${\bar{K}}_{i2}$ values, finally, are very close to each other, their values being within the range of the concentrations that were employed in the present experiments and their standard deviations being acceptable if one considers the complexity of the underlying phenomena. It should be noted that the similarity of the ${\bar{K}}_{i1}$ and ${\bar{K}}_{i2}$ values (they differ by only 3.4%) allows classification of the inhibition caused by the condensed tannin of the *pinhão* coat tannins as of the simple parabolic non-competitive type [13,14]. In this case, the value of the inhibition constants becomes also the approximate concentration of the condensed tannin that produces 50% inhibition at a fixed substrate concentration, *i.e.*, [I]_50_ ≈ ${\bar{K}}_{i1}$ ≈ ${\bar{K}}_{i2}$. This postulate can be easily tested by examining the reaction rate *versus* inhibitor concentration curve in Figure 1A. Numerical interpolation yielded 316.3 μg/mL as the concentration producing 50% inhibition, a value that is very close to that predicted by the optimized inhibition constants.

The substrate inhibition caused by *p*-nitrophenyl palmitate was not very pronounced, as revealed by comparison of the K_M_ and K_iS_ values, the latter exceeding the former by a factor of 24.4 (see Table 1). However, it was consistently present in the four experimental curves that were measured in the present work, and if neglected, it would have interfered significantly with the interpretation of the results. It must be mentioned, however, that substrate inhibition is a frequently-reported phenomenon with pancreatic lipase [16], as well as with other lipases [17]. It should be noted, on the other hand, that the general kinetics and K_M_ values of lipases can be expected to vary considerably depending on the way in which the lipophilic substrate is solubilized and the general conditions of the assay. A K_M_ of 2.7 μM has been reported, for example, for pancreatic lipase hydrolyzing the same substrate used in the present work, but in a medium containing 5 mM sodium deoxycholate in addition to 10% isopropanol and 50 mM phosphate buffer at pH 8.0 [18].

**Table 1:** Kinetic parameters of porcine pancreatic lipase obtained by fitting Equations (2) and (3) to the experimental data. Both equations were fitted simultaneously to the four substrate saturation curves obtained with the different *pinhão* coat extract concentrations shown in Figure 2. A non-linear least-squares procedure was used. The error terms correspond to standard deviations of the optimized parameters. The model selection criterion was 4.448, the correlation coefficient 0.995 and the sum of squared deviations 0.0541.

Parameters	Optimized Values	
	Equation (2)	Equation (3)
K_M_ (mM)	0.0583 ± 0.0077	0.0581 ± 0.0068
V_max_ (μmol·min^−1^)	2.094 ± 0.126	2.079 ± 0.112
K_iS_ (mM)	1.401 ± 0.337	1.418 ± 0.311
K_i1_ (μg·mL^−1^)	97,620 ± 15,080,211	—
$K_{i1}^{\prime }$ (μg·mL^−1^)	1.090 ± 168.991	—
K_i2_ (μg·mL^−1^)	1604.5 ± 1887.5	—
$K_{i2}^{\prime }$ (μg·mL^−1^)	82.19 ± 121.9	—
${\bar{K}}_{i1}$ (μg·mL^−1^)	—	332.7 ± 146.1
${\bar{K}}_{i2}$ (μg·mL^−1^)	—	321.2 ± 93.0
Sum of squared deviations	0.0614	0.0541
Model selection criterion	4.210	4.448

### 3.3. Effects on Triglyceride Absorption

**Figure 3:**
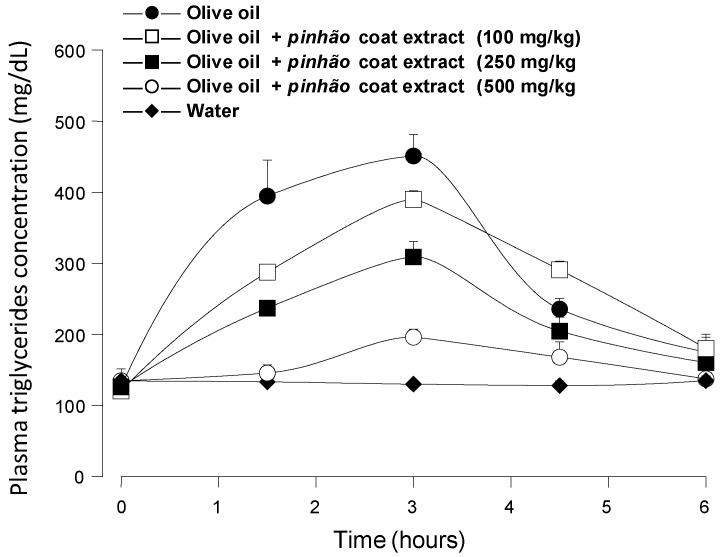
Blood triglyceride concentration profiles after intragastric olive oil loads in mice: the effect of the *pinhão* coat extract. The oral administration of olive oil was done immediately after the oral administration of three different doses of the *pinhão* coat extract. The doses that were administered are given on the graphs. Plasma triglycerides were measured as described in the Experimental Section. Each value represents the mean ± SD of three mice.

**Figure 4:**
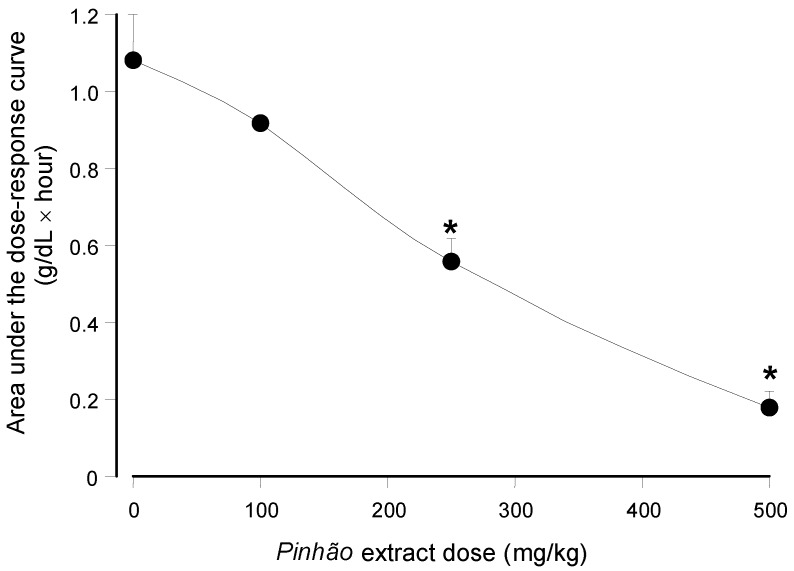
Dependence of the area under the plasma triglyceride concentration *versus* time on the administered *pinhão* coat extract dose. The areas were obtained from the results shown in Figure 3. Each value represents the mean ± SD of three mice. Asterisks indicate statistical significance relative to the control (*p* ≤ 0.05 according to the Student–Newman–Keuls test).

The ability of the *pinhão* coat extract rich in tannins as an inhibitor of pancreatic lipase seems to be well demonstrated by the *in vitro* experiments. Confirmation of its effectiveness on the triglyceride hydrolysis *in vivo* can be obtained by means of experiments in which the blood levels of triglycerides are measured after an oral administration of these lipids. This test is based on the well-established notion that triglyceride hydrolysis is essential for its effective intestinal absorption along a mechanism that involves hydrolysis and re-esterification [8]. Figure 3 shows the results of the experiments in which an oral load of olive oil was applied to mice alone or in combination with three different doses of the *pinhão* coat extract, namely 100 mg/kg, 250 mg/kg and 500 mg/kg. The triglyceride levels were monitored during 6 h after administration. When water was given, the triglyceride levels remained more or less constant at around 115 mg/dL, providing a convenient base line for the 6-h period. Administration of olive oil alone caused a substantial rise in the plasma triglyceride levels, which reached values slightly above 400 mg/dL after 3 h. After six hours, they had almost declined to the basal levels. The triglyceride levels were reduced upon the *pinhão* coat extract administration, the effect being a function of the dose that was administered. This effect *versus* dose relationship can be seen in Figure 4 in which the areas under the dose-response curves were plotted against the *pinhão* extract doses. The base-line for calculating the areas under the curves in Figure 4 was provided by the plasma triglyceride levels measured after water administration (Figure 3). These areas have the dimensions of (g/dL × h), and they are a function of the lipid load that was effectively absorbed by the intestine. Figure 4 shows that the areas under the curves declined almost linearly with the extract dose that was administered. At the highest dose (500 mg/kg), it was diminished by 84% relative to the control (no extract administration); a 50% reduction can be expected at a dose around 258.9 mg/kg.

**Figure 5:**
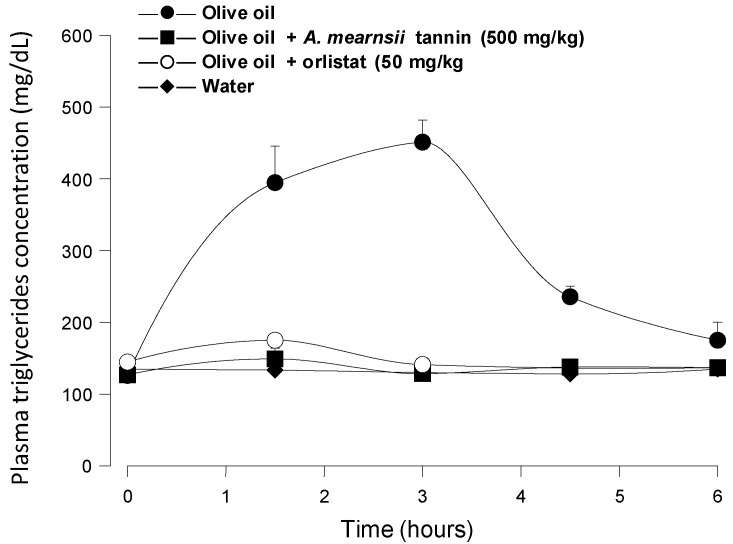
Blood triglyceride concentration profiles after intragastric olive oil loads in mice: The effects of orlistat and *A. mearnsii* tannin. The oral administration of olive oil was done immediately after the oral administration of orlistat and *A. mearnsii* tannin.The doses that were administered are given in the graphs. Plasma triglycerides were measured as described in the Experimental Section. Each value represents the mean ± SD of three mice.

Figure 5 shows the results of positive control experiments in which orlistat (50 mg/kg) and *A. mearnsii* tannin (500 mg/kg) were administered to mice in the same manner as the *pinhão* coat extract. It is apparent that both orlistat (a classical lipase inhibitor) and the *A. mearnsii* tannin were quite effective at diminishing triglyceride absorption, the area under the corresponding curves representing less than 5% of the area under the control curve. This is not surprising for orlistat, which is a covalent inhibitor of pancreatic lipase [7], but is not necessarily expected from the *A. mearnsii* tannin, which is a much weaker lipase inhibitor than the *pinhão* coat tannin (see Figure 1). It should be mentioned that in this respect, the commercial *A. mearnsii* tannin preparation used in the present work was even more effective than the *A. mearnsii* polyphenol preparation used in previous studies [7], which diminished by 65% the area under the curve when a 500-mg/kg dose was administered in experiments similar to those of the present work. The difference between the *pinhão* coat tannin and the *A. mearnsii* tannin preparation in affecting triglyceride absorption may have several causes. The *pinhão* coat extract is certainly a better inhibitor of the *p*-nitrophenyl palmitate hydrolysis than the *A. mearnsii* tannin under the conditions of our assay. The latter, however, was done in the presence of isopropanol and with a non-natural substrate (*p*-nitrophenyl-palmitate). One cannot exclude that, under physiologic conditions, with the natural substrates (long-chain triglycerides)) and in the presence of bile acids and colipase, the *A. mearnsii* tannin reveals itself to be a better inhibitor than the *pinhão* coat extract. It should also be considered that the enzyme used in the *in vitro* assays was obtained from pig pancreas, and the *in vivo* experiments were done with mice. The actions of both the *A. mearnsii* tannin and the *pinhão* coat extract on both enzymes can be different. Finally, it is possible that the mouse responds differently to both preparations. This includes different gastric movements, but also the possibility that the *A. mearnsii* tannin site of action is not restricted to pancreatic lipase. Irrespective of the real reason for the discrepancy, a definitive explanation depends on additional conclusive evidence.

A mechanism for the anti-obesity actions of an *A. mearnsii* polyphenol preparation has been proposed as consisting of a combination of lipase inhibition and maltase and sucrase inhibition [7]. The first action reduces triglyceride absorption and the second sugar absorption. A similar anti-obesity action can be proposed for the *pinhão* coat tannin based on the results obtained in the present work and on the inhibitory actions of the same preparation on both salivary and pancreatic α-amylases and on starch absorption that have been demonstrated in a previous work [5]. It remains to be investigated if absorbable constituents of the *pinhão* coat extract are able to alter the expression of obesity and diabetes-related genes in the adipose tissue, muscle and liver, as demonstrated to occur when mice are treated with an *A. mearnsii* polyphenol preparation [19]. On the other hand, it is important to emphasize that the actions demonstrated in the present work for the *pinhão* coat extract are similar not only to those proposed for the *A. mearnsii* polyphenols [7,19], but also for analogous constituents of other plants, such as apples [6] and several types of tea [20,21].

Inhibition of pancreatic lipase, as well as of α-amylases by the *pinhão* coat tannin requires binding to these enzymes, a phenomenon that was clearly demonstrated to exist for several combinations of tannins and proteins, including enzymes [22,23,24]. Binding of tannins to proteins involves both hydrophilic and hydrophobic interactions. It is non-specific in some cases and specific with a certain degree of cooperativity in others [22]. In the case of the *pinhão* coat condensed tannin, where procyanidins predominate [5], binding is a complex phenomenon, as indicated by the parabolic inhibition kinetics and probably facilitated by the numerous hydroxyl groups. These groups are probably responsible for the most important interactions at low concentrations [24]. At high concentrations, however, random hydrophobic stacking of the planar rings may occur between tannin and protein, as deduced for the wine tannins binding to saliva proteins based on nuclear magnetic resonance and molecular modeling [24].

## 4. Conclusions

The results obtained in the present study revealed that the *pinhão* coat tannin is an effective inhibitor of pancreatic lipase. Consistently, it was also effective at diminishing the plasma triglyceride levels in mice after a load of olive oil. This is most probably the consequence of an indirect inhibition of triglyceride absorption via inhibition of pancreatic lipase [8]. For the *pinhão* coat tannin, this is the second report of a biological activity, the first one being a similar inhibition of the absorption of glucose derived from starch as a consequence of an inhibitory action on α-amylase [5]. All of these actions are compatible with a potential anti-obesity action, as suggested for other polyphenol or tannin-rich preparations [5,6,20,21].
